# Clinical validation of a highly sensitive assay to detect EGFR mutations in plasma cell-free DNA from patients with advanced lung adenocarcinoma

**DOI:** 10.1371/journal.pone.0183331

**Published:** 2017-08-22

**Authors:** Yuping Li, Hanyan Xu, Shanshan Su, Junru Ye, Junjie Chen, Xuru Jin, Quan Lin, Dongqing Zhang, Caier Ye, Chengshui Chen

**Affiliations:** 1 Department of Pulmonary and Critical Care Medicine, The First Affiliated Hospital of Wenzhou Medical University, Wenzhou, Zhejiang, China; 2 Department of Radiology, The First Affiliated Hospital of Wenzhou Medical University, Wenzhou, Zhejiang, China; Baylor College of Medicine, UNITED STATES

## Abstract

**Background:**

Circulating tumor DNA (ctDNA) is a promising biomarker for noninvasive epidermal growth factor receptor (EGFR) mutations detection in lung cancer patients, but the existing methods have limitations in sensitivity or in availability. In this study, we evaluated the performance of a novel assay called ADx-SuperARMS in detecting EGFR mutations in plasma cell-free DNA from patients with advanced lung adenocarcinoma.

**Methods:**

A total of 109 patients with metastatic advanced adenocarcinoma were recruited who provided both blood samples and matched tumor tissue samples. EGFR mutation status in plasma samples were tested with ADx-SuperARMS EGFR assay and tumor tissue samples were tested with ADx-ARMS EGFR assay. The clinical sensitivity, specificity, positive prediction value (PPV), and negative prediction value (NPV) of ADx-SuperARMS EGFR assay were calculated by using EGFR mutation status in tumor tissue as standard reference. A receiver operating characteristic (ROC) analysis was implemented and an area under the curve (AUC) was calculated to evaluate sensitivity and specificity of exon 19 deletion (E19Del) and L858R mutation detection. The objective response rate (ORR) were calculated according to the EGFR mutation status determined by ADx-superARMS as well.

**Results:**

0.2% analytical sensitivity and 100% specificity of the ADx-SuperARMS EGFR assays for EGFR E19Del, L858R, and T790M mutants were confirmed by using a series of diluted cell line DNA. In the clinical study, EGFR mutations were detected in 45.9% (50/109) of the plasma samples and in 56.9% (62/109) of the matched tumor tissue samples. The sensitivity, specificity, PPV and NPV of the ADx-SuperARMS EGFR assay for plasma EGFR mutation detection were 82.0% (50/61), 100% (48/48), 100% (50/50), and 81.4% (48/59), respectively. In ROC analysis, ADx-SuperARMS achieved sensitivity and specificity of 88% and 99% in E19Dels as well as sensitivity and specificity of 89% and 100% in L858R, respectively. Among the 35 patients who were plasma EGFR mutation positive and treated with first generation of EGFR-tyrosine kinase inhibitors (TKIs), 23 (65.7%) achieved partial response, 11 (31.4%) sustained disease, and 1 (2.9%) progressive disease. The ORR and disease control rate (DCR) were 65.7% and 97.1%, respectively.

**Conclusions:**

ADx-SuperARMS EGFR assay is likely to be a highly sensitive and specific method to noninvasively detect plasma EGFR mutations of patients with advanced lung adenocarcinoma. The EGFR mutations detected by ADx-SuperARMS EGFR assay could predict the efficacy of the treatment with first generation of EGFR-TKIs. Hence, EGFR blood testing with ADx-SuperARMS could address the unmet clinical needs.

## Introduction

Lung cancer has the highest incidence among all cancers and is the leading cause of death worldwide[1, 2]. Non-small cell lung cancer (NSCLC) is the predominant type of the disease, accounting for approximately 85% of all lung tumors[3, 4]. The majority of patients present with advanced disease, and chemotherapy has long been the standard treatment as the first-line therapy for patients with advanced NSCLC[5]. In recent years, epidermal growth factor receptor-tyrosine kinase inhibitors (EGFR-TKIs) including Gefitinib, Erlotinib, Icotinib, and Afatinib have been successfully developed and demonstrated much higher response rate and less side effect than chemotherapy in the treatment of patients with advanced NSCLC with sensitizing EGFR mutations[6–10]. Therefore, EGFR mutation detection to select proper patients for EGFR-TKI treatment is critical for the clinical practice[11, 12]. Currently, the tumor tissue has been used as the standard sample type for EGFR mutation detection in the clinical practice, but it has several limitations. Firstly, the tumor tissue is unavailable for some patients, especially for those with a recurrent tumor[13]. Secondly, the intra-tumor heterogeneity makes the mutation detection of the tumor tissue at a risk of sampling bias[14]. Last but not the least, the availability of 3rd generation of EGFR-TKIs such as Osimertinib which is specially targeting acquired resistant mutation T790M triggers the realistic clinical need of dynamic monitoring of EGFR mutation[15, 16], but it is a challenge for patients to take an invasive re-biopsy for a series of mutation testings[17, 18]. Therefore, circulating tumor DNA (ctDNA)-based noninvasive tumor mutation detection has been applied to the clinical practice[19–27]. For the past decade, several key methods have been developed to detect EGFR mutations with ctDNA in plasma samples, such as direct sequencing, amplification-refractory mutation system (ARMS), droplet digital PCR (ddPCR), next generation sequencing (NGS), denaturing high-performance liquid chromatography (DHPLC) and so on[23, 24, 28–38]. Among them, ARMS is considered as an economical, easy-to-take and fast method, and therefore it has been extensively used for the clinical practice as well as clinical trials of target therapies. Nevertheless, when testing EGFR mutations in the plasma ctDNA, the sensitivity of ARMS was only 48.2–67.4% compared to the matched tumor tissue, in spite of a high specificity of 93.5–99%[29,35, 39–41], which indicates that the blood-based EGFR mutation testing method is limited by a relatively low sensitivity. Therefore, an improved method convenient as ARMS with a higher sensitivity is desired. Most recently, a newly developed EGFR mutation assay called ADx-SuperARMS has been available for detecting EGFR mutation status in the plasma ctDNA, which is claimed to be much more sensitive than ARMS. In the present study, we implemented a clinical study to evaluate the performance of this new method in testing clinical plasma samples as well as in predicting the efficacy of EGFR-TKIs treatment.

## Materials and methods

### Patients

This study was conducted in the Department of Pulmonary and Critical Care Medicine, the first affiliated hospital of Wenzhou Medical University in Zhejiang, China. Patients were enrolled from 1 August 2016 to 1 January 2017. Inclusion criteria were as below: 1) pathologically confirmed lung adenocarcinoma; 2) advanced clinical stage (stage IIIB or IV) according to the seventh edition of the cancer staging system[42]; 3) newly diagnosed or progressive disease (PD) after EGFR-TKIs treatment or recurrence after surgery; 4) a subsequent treatment did not begin at the time of sample collection; 5) valid tissue and blood specimens available and the interval between tissue collection and blood collection was less than 14 days.

The study protocol was approved by the Institutional Review Board of the first affiliated hospital of Wenzhou Medical University. All patients provided written informed consent before the enrollment.

### Specimen collection and DNA extraction

Samples of tumor tissue and matched peripheral whole blood were collected from each patient before the subsequent treatment. All tumor tissue samples were formalin-fixed, paraffin-embedded (FFPE) diagnostic samples. Four to eight sections (5um thickness) of tumor tissue samples were used for DNA extraction by using QIAamp DNA FFPE Tissue Kit (Qiagen, Hilden, Germany) according to the manufacturer’s instructions. Each peripheral whole blood sample of 10mL from each patient was collected into EDTA tube and centrifuged within 1 hour at room temperature at 2000g for 10 min first, and then 8000g for 10 min to isolate the plasma, which was then stored at -80°C until cell-free DNA (cfDNA) extraction. Plasma cfDNA was extracted from 4 mL of plasma from each patient with the QIAamp Circulating Nucleic Acid kit (Qiagen, Hilden, Germany) following the manufacturer’s instructions. Eluted DNA was used to detect EGFR mutations immediately.

### Detection of EGFR mutations in tumor tissue by ADx-ARMS EGFR kit

EGFR mutations in tumor tissue samples were detected by using ADx-ARMS EGFR kit (Amoy Diagnostics, Xiamen, China) that covers the 29 most common somatic EGFR mutations (S1 Table). Experimental procedure and data analysis followed the manufacturer's instructions.

### Detection of EGFR mutations in plasma ctDNA by ADx-SuperARMS EGFR kit

EGFR mutations in plasma samples were detected by using ADx-SuperARMS EGFR mutation detection kit (Amoy Diagnostics, Xiamen, China), which can identify 41 types of the most common somatic EGFR mutations in exons 18-21(S2 Table). Experimental procedure and genotype calling followed the manufacturer's instructions: Thaw the P-EGFR Positive Control and P-EGFR Reaction Mix at room temperature, mix the reagents by inverting the tube 10 times and centrifuge briefly to collect the contents at the bottom of the tube, briefly centrifuge P-EGFR Enzyme Mix prior to use. Add 67.5 μL DNA sample and 2.16 μL P-EGFR Enzyme Mix into 247.5 μL P-EGFR Reaction Mix. Add 67.5 μL no-template controls (sterile water, NTC) and 2.16 μL P-EGFR Enzyme Mix into 247.5 μL P-EGFR Reaction Mix. Add 67.5 μL P-EGFR Positive Control and 2.16 μL P-EGFR Enzyme Mix into 247.5 μL P-EGFR Reaction Mix. Mix each solution thoroughly by gently pipetting up and down more than 10 times, then centrifuge briefly. Take out the P-EGFR Reaction Strips and centrifuge the strips if there are any reagent droplets in the caps of the PCR tubes, then briefly uncover the caps prior to use. Transfer 70 μL of above mixed solution to the appropriate PCR tube of the P-EGFR Reaction Strips and seal the strips. Spin down the PCR tubes gently or centrifuge them briefly to collect the reagents at the bottom of tubes. Place the PCR tubes into the real-time PCR instrument. PCR reactions were carried out on Mx3000P qPCR System (Agilent) under a thermal profile as follows: incubation at 95°C for 10 min, followed by 15 cycles of 95°C for 40 seconds, 64°C for 40 seconds and 72°C for 30 seconds, and then 28 cycles of 93°C for 40 seconds, 60°C for 45 seconds and 72°C for 30 seconds. The raw data were processed by the software bundled with the instrument, and ΔCt was calculated as: ΔCt = mutant Ct value—internal control Ct value. If the ΔCt value is less than the corresponding threshold, the sample is defined as positive. Otherwise, the sample is classified as negative or below the detection limit of the kit.

### Statistical analysis

The Fisher's exact test was implemented to assess the relationship between positive rates in matched tissue and plasma samples. A two-sided P-value of <0.05 was considered as a statistical significance. EGFR mutation status in the tumor tissues were used as standard reference for the calculation of the concordance, sensitivity and specificity of plasma EGFR mutation detection. The concordance rate was calculated as the sum of the positives and the negatives in both samples, divided by the total number of matched samples. The sensitivity was calculated as the proportion of the concordant positives in both samples out of the positive tissue samples, whereas the specificity was calculated as the proportion of the concordant negatives in both samples out of the negative tissue samples. A receiver operating characteristic (ROC) analysis was implemented and an area under the curve (AUC) was calculated to evaluate sensitivity and specificity of exon 19 deletion (E19Del) and L858R mutation detection. The maximal sensitivity and specificity in ROC analysis were determined by the Youden’s index, namely sensitivity + specificity − 1. To assess the value of EGFR mutations in ctDNA in predicting efficacy of the first-generation EGFR-TKIs, the objective response rate (ORR) and the disease control rate (DCR) were measured according to the Response Evaluation Criteria In Solid Tumors version 1.1[43], where ORR was defined as the rate of the patients with complete response (CR) and partial response (PR) after EGFR-TKIs therapy and DCR was defined as the rate of patients with CR, PR and sustained disease (SD) after EGFR-TKIs therapy.

## Results

### Characteristics of patients

A total of 109 patients met the criteria and were prospectively enrolled into the study. The patients’ clinical characteristics are listed in Table 1. The patients consisted of 58 women and 51 men. 36 patients were smokers, and 73 were never-smokers. Five patients previously underwent surgery for early-stage tumors, 4 patients were recurrent ones (progressive disease) from previous treatment of first generation of EGFR-TKIs, and the remaining 100 patients were newly diagnosed with treatment naïve at stages of IIIB to IV lung adenocarcinoma. Twenty (18.3%) patients were classified as at stage IIIB, and 89 (81.7%) at stage IV. The ways for tumor tissue collection included: primary cancer via transbronchial lung biopsy for 22 patients, pulmonary aspiration for 60, pleural effusion cell block for 11, resected intraoperatively for 1, and metastatic sites for 15 (7 from lymph nodes and 8 from pleura). All specimens were confirmed to be lung adenocarcinoma by pathologists.

**Table 1:** Clinical characteristics of 109 patients with lung adenocarcinoma.

Characteristic	No. of Patients(N = 109)	Percentage(%)
Age, years		
<60	31	28.4
≥60	78	71.6
Sex		
Male	51	46.8
Female	58	53.2
Stage		
IIIB	20	18.3
IV	89	81.7
Smoking history		
Never smokers	73	67.0
Current or former	36	33.0
Stage of treatment		
Newly diagnosed	100	91.7
PD after EGFR-TKIs	4	3.7
Recurrence after surgery	5	4.6
Tissue		
Biopsy under bronchoscopy	22	20.2
Pulmonary aspiration	60	55.1
Intraoperatively	1	0.9
Lymph nodes	7	6.4
Pleura	8	7.3
Pleural effusion cell blocks	11	10.1

PD: progressive disease; EGFR-TKIs: epidermal growth factor receptor-tyrosine kinase inhibitors.

### EGFR mutations in tumor tissues and matched plasma samples

In the total 109 tumor tissues, 62 (56.9%) samples harbored EGFR mutations, of which 31(50.0%) samples carried single EGFR E19Del, 23 (37.1%) single L858R mutation, 1(1.6%) G719X mutation, 1 (1.6%) exon 20 insertion (E20-ins) mutation and 6 (9.7%) double mutations (1 had both E19Dels and L858R, 3 had both L858R and T790M, 2 had both E19Dels and T790M). E19Del was the most common EGFR mutation and L858R was the second. The proportion of other EGFR mutation subtypes such as G719X and E20-ins was low.

In the matched plasma samples, the EGFR mutations positive rate was 45.9% (50/109), which is similar to what in tumor tissue (P = 0.1359). Of the 50 EGFR mutation positive plasma samples, 25 (50.0%) harbored single E19Dels, 21 (42.0%) single L858R mutation, 1 (2.0%) E20-ins mutation and 3 (6.0%) double mutations (2 E19Del + T790M, 1 L858R + T790M), suggesting a similar mutation profiling to the tumor tissues. Twelve patients were detected to be EGFR mutation positive in tumor tissues but negative in plasma samples (S3 Table). Of these mutations in tumor tissues, 6 were E19Del, 3 L858R, 1 G719X, and 2 L858R+T790M. Interestingly, another three cases were determined as EGFR mutation positives in both plasma and tumor tissue samples with inconsistent mutation subtypes: 1 with L858R only in plasma but L858R+T790M in tumor tissue, 1 with L858R only in plasma but L858R + E19Del in tumor tissue, and 1 with L858R + T790M in plasma but L858R only in tumor tissue. The details of mutation status in tissue and plasma samples are shown in S3 Table. Of note, the case with G719X mutation in tumor tissue were further determined as G719S by next generation sequencing (NGS). Since the EGFR mutation subtype (G719S) was not covered by the current ADx-SuperARMS EGFR assay, this case with G719X mutation was defined as a wild type in both plasma and tumor tissue in the following comparisons and therefore the total inconsistent cases between the paired samples were eleven (Table 2). Besides, as for the 4 cases with acquired EGFR-TKI resistance, two of them were E19Del+T790M positive both in tumor tissue and in plasma, one was L858R+T790M positive in tumor tissue but single L858R positive in plasma, and one was single L858R positive in tumor tissue but L858R+T790M positive in plasma.

**Table 2:** Comparison of EGFR mutation status in matched plasma and tissue samples (N = 109).

		Tumor Tissue		Total
		Mt	Wt	
Plasma	Mt	50	0	50
	Wt	11	48	59
	Total	61	48	109
Sensitivity*	82.0% (72.3%-91.6%)			
Specificity*	100% (100.0%-100.0%)			
PPV*	100.0% (100.0%-100.0%)			
NPV*	81.4% (71.4%-91.3%)			
Overall agreement*	89.9% (84.3%-95.6%)			

*percentage [95% confidence interval (CI)];

Mt: mutation; Wt: wild type; PPV: positive predictive value; NPV: negative predictive value

### The sensitivity and specificity of ADx-SuperARMS EGFR assay in detecting plasma EGFR mutation

Firstly, the analytical sensitivity and specificity of the ADx-SuperARMS assays for EGFR E19Dels, L858R, and T790M mutations were evaluated by using a series of mock DNA samples (S1 File). As the mutant percentage went from 5% down to 0.1%, the mean ΔCt values of the three subtypes of EGFR mutations, namely E19Dels, L858R, and T790M, ranged from 6.3 to 11.6, from 6.4 to 11.4 as well as from 2.7 to 10.8, respectively (Table 3). With the thresholds of the positive callings recommended in the manufacturer’s instruction, we obtained true positives for all three EGFR mutations subtypes at a mutant level of 0.2% or more, suggesting a detection limitation of 0.2% for EGFR mutations. Of note, when no mutant DNA inputted, no signal was detected, which indicated a specificity of 100%. Therefore, an analytical sensitivity of 0.2% and a specificity of 100% for all three major EGFR mutation subtypes were achieved.

**Table 3:** Verification results of the ADx-SuperARMS EGFR assays.

Mutation	ΔCt value cut-off for positive calling	Mean ΔCt value from 0% of mutant in total DNA	Mean ΔCt value from 5% of mutant in total DNA	Mean ΔCt value from 1% of mutant in total DNA	Mean ΔCt value from 0.2% of mutant in total DNA	Mean ΔCt value from 0.1% of mutant in total DNA
E19Del	11	No ΔCt	6.3	8.8	10.6	11.6
L858R	11	No ΔCt	6.4	8.1	10.7	11.4
T790M	8	No ΔCt	2.7	4.4	7.2	10.8

EGFR, epidermal growth factor receptor; E19Del: exon 19 deletion.

**Fig 1:**
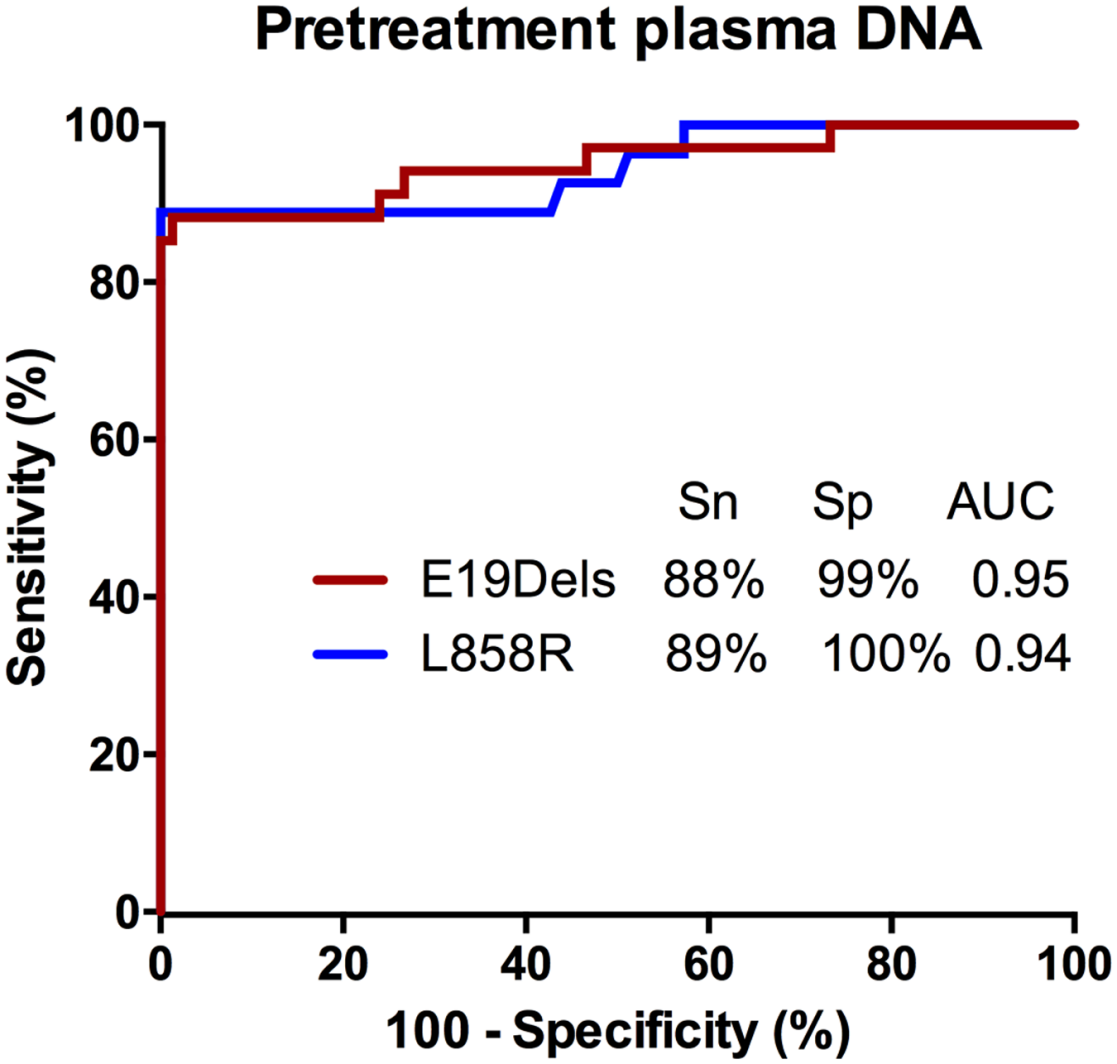
Sensitivity and specificity assessment with ROC analysis in plasma ctDNA from pretreatment samples. E19Del: exon 19 deletion; Sn, sensitivity; Sp, specificity; AUC, area under the curve.

Next, we assessed the clinical sensitivity and specificity of ADx-SuperARMS EGFR assay in detecting plasma EGFR mutation. In the 109 tumor tissue-plasma paired clinical samples, 98 carried the same EGFR mutation status in both samples, giving a concordance of 89.9% (Table 2). Eleven cases were determined as negative in plasma but positive in tumor tissue, whereas no sample was mutant in plasma but wild type in tissue. Compared to the matched tumor tissues, the sensitivity and specificity for EGFR mutation detection in plasma by ADx-SuperARMS were 82.0% (50/61) and 100% (48/48), respectively. The positive predictive value (PPV) was 100% (50/50), and the negative predictive value (NPV) was 81.4% (48/59). The concordance between plasma and tumor tissue was 89.9% (Table 2). Furthermore, a ROC analysis was applied for E19Dels and L858R mutation detection in plasma, and ADx-SuperARMS achieved an AUC of 0.95 with maximal sensitivity and specificity of 88% and 99% in E19Dels as well as an AUC of 0.94 with maximal sensitivity and specificity of 89% and 100%, respectively (Fig 1).

It is reported that the false positive rate is as high as 48.5% when detecting EGFR mutation in FFPE tumor tissue samples with ultra-sensitive method, especially for T790M[44], so no comparison between the performances of ADx-SuperARMS in tumor tissue versus in plasma was implemented.

### EGFR mutations in plasma for predicting efficacy of EGFR-TKIs treatment

**Fig 2:**
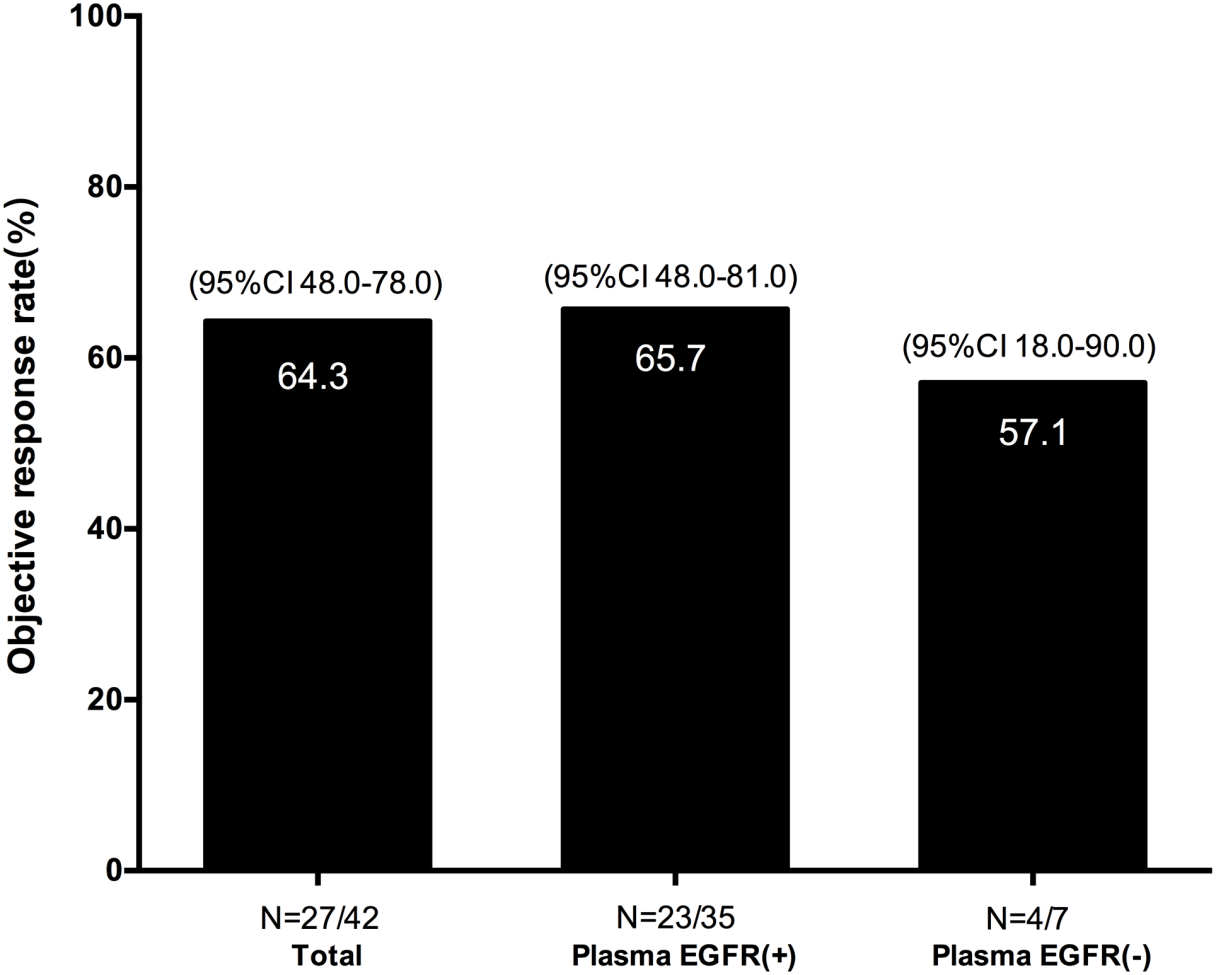
Objective response rate according to EGFR mutation status in patients who were plasma EGFR mutation positive and plasma EGFR mutation negative. EGFR, epidermal growth factor receptor; CI, confidence interval.

Out of the 109 patients, 42 (including 41 newly diagnosed and 1 recurrent disease after surgery) had measurable tumors and positive EGFR mutation in tumor tissues and received Gefitinib or Icotinib as first-line treatment. Up to May 3, 2017, 27 (64.3%) patients achieved a PR, 14 (33.3%) SD, and 1 (2.4%) PD, according to the Response Evaluation Criteria in Solid Tumors 1.1 (RECIST). The ORR and DCR were 64.3% (27/42) and 97.6% (41/42), respectively. Taking the plasma EGFR mutation status into account, we observed an ORR of 65.7% (23/35) and a DCR of 97.1% (34/35) in plasma EGFR mutation positive patients. In contrast, an ORR of 57.1% (4/7) was observed in plasma EGFR mutation negative but tumor tissue EGFR mutation positive patients (Fig 2). The EGFR-TKI treatment outcomes of patients with EGFR mutation positive in plasma tended to be better than the negative ones, in spite of no significant difference (65.7% vs. 57.1%, P = 0.6858).

## Discussion

In clinical practice, EGFR mutation assessment is recommended to guide target therapy for NSCLC patients, and ctDNA is a promising biomarker for noninvasive EGFR mutation detection as well as other molecular testing for personalized cancer management[19–27]. However, the existing methods for ctDNA EGFR mutation detection were either limited by relatively low sensitivity such as ARMS, or barely used in the clinical practice due to complicated procedure, long turn-around time or unavailability of the equipment, such as droplet digital PCR (ddPCR) and Next Generation Sequencing (NGS) [23, 24, 28–38]. Hence, a widely available, easy-to-operate and sensitive method for EGFR mutation detection in plasma ctDNA is urgently desired for the routine clinical use.

In this study, we presented ADx-SuperARMS as a highly sensitive technology to improve PCR-based ctDNA EGFR mutation detection. Using a series of diluted cell line DNA, we validated its analytical sensitivity of 0.2% under the specificity of 100% for three major mutation subtypes, which is a great improvement of traditional ARMS method[45, 46]. Next, in our clinical study, we observed a high clinical sensitivity (82.0%) and specificity (100%) of ADx-SuperARMS EGFR mutation blood testing. E19Dels and L858R were the two most common EGFR mutation subtypes in our study, just as reported in previous Chinese NSCLC studies[47, 48]. Compared to the performance of ddPCR[30], ADx-SuperARMS provides a better sensitivity of E19Dels (88% vs. 82%) and L858R (89% vs. 80%) mutation detection in plasma with a specificity of 99% and 100% respectively. Our findings indicated that ADx-SuperARMS is competent for noninvasive detection of clinically actionable cancer mutations to guide the target therapy, especially when the tumor tissue is not available Furthermore, as a PCR-based method, ADx-SuperARMS is more convenient, more economical and easier to set up than other highly sensitive blood testing assays, like ddPCR and NGS.

In our study, three out of five patients with multiple EGFR mutations exhibited inconsistent mutation subtypes between tumor tissue and matched plasma samples, which was also observed in previous studies[19, 28–30]. Out of the three, two had more mutation subtypes (E19Del + L858R or L858R + T790M) detected in tissue than in matched plasma (L858R), which is partially because a minority of EGFR mutated tumor clone release few or even no mutant DNA fragments into the circulation so that no mutation could be detected from circulating cell-free DNA templates. In contrast, one patient had more mutation subtypes (L858R + T790M) detected in plasma than in tumor tissue (L858R). One possible reason for this inconsistency is the intra-tumor heterogeneity of genetic abnormalities, and the good performance of ADx-SuperARMS shed a light on addressing the tumor heterogeneity with ctDNA testing.

Molecular testing of ctDNA is reported to provide a dynamic monitoring of acquired resistance to first generation EGFR-TKIs[49–53]. In our study, all the four cases with acquired resistance to EGFR-TKI harbored T790M in tissue or in plasma or both (2 patients had T790M detected in both tissue and plasma, 1 in tissue and 1 in plasma). Noticeably, in spite of the small sample size of EGFR-TKI resistance, three out of four drug resistant patients turned out to be T790M ctDNA carriers, including one undetected in tissue. Our findings suggest that blood testing with ADx-SuperARMS is feasible for noninvasively monitoring of emergent secondary mutation related to the acquired resistance during target therapy. Additional clinical study is ongoing on more patients who have progressed disease after EGFR-TKIs treatment.

Previous studies reported significant correlations between ctDNA EGFR mutation status and the clinical response to EGFR-TKIs[23, 24, 29]. Here in our study, the ORR of EGFR-TKIs treatment in plasma EGFR mutation positive patients was very close to the ORR in tissue EGFR mutation positive patients (65.7% vs. 64.3%), and it was better than in plasma EGFR negative cohort (65.7% vs 57.1%, Fig 2), suggesting that the blood testing with ADx-SuperARMS could predict the patients’ benefit from EGFR-TKIs treatment. The progression-free survival and overall survival of patients during EGFR-TKIs treatment have not been achieved and the clinical follow-up observation is still ongoing.

In conclusion, ADx-SuperARMS is likely to provide a highly sensitive and specific noninvasive detection of EGFR mutations in clinical blood samples from advanced lung adenocarcinoma patients to guide target therapy as well as to monitor drug resistance, and the EGFR mutation status detected in plasma with ADx-SuperARMS could predict the efficacy of EGFR-TKIs treatment. Hence, EGFR blood testing with ADx-SuperARMS offers a good option to address the unmet clinical needs. As the study cohort is relatively small in this study, further investigation of larger patient population is needed to confirm the findings from this study.

## Supporting information

S1 Table Details of 29 somatic mutations types in EGFR gene in the ADx-ARMS kit. EGFR, epidermal growth factor receptor; Ex: exon.
(DOCX)

S2 Table Details of 41 somatic mutations types in EGFR gene in the ADx-SuperARM kit. EGFR, epidermal growth factor receptor; Ex: exon.
(DOCX)

S3 Table EGFR mutation status in plasma and matched tumor tissue samples. EGFR, epidermal growth factor receptor; E19Del: exon 19 deletion.
(DOCX)

S1 File Assessing the analytical sensitivity and specificity of the ADx-SuperARMS EGFR assay. (DOCX)

S1 STROBE Checklist STROBE Statement—Checklist of items that should be included in reports of observational studies. (DOCX)
