# Correction: Clinical validation of a highly sensitive assay to detect EGFR mutations in plasma cell-free DNA from patients with advanced lung adenocarcinoma

**DOI:** 10.1371/journal.pone.0189549

**Published:** 2017-12-07

**Authors:** The PLOS ONE Staff

There is an error in the XML that is causing the designation of the Corresponding Author to be indexed incorrectly. Chengshui Chen should be designated as the Corresponding Author, and his email is: wzchencs1@163.com. The publisher apologizes for the error.
