# Supplementary material for: Clinical validation of a highly sensitive assay to detect EGFR mutations in plasma cell-free DNA from patients with advanced lung adenocarcinoma
Source: PLoS One. 2017 Aug 22;12(8):e0183331. doi: 10.1371/journal.pone.0183331 (PMC5568724; doi:10.1371/journal.pone.0183331)
Supplement: S2 Table — EGFR, epidermal growth factor receptor; Ex: exon. (DOCX) [file pone.0183331.s002.docx]

S2 Table Details of 41 somatic mutations types in EGFR gene in the ADx-SuperARMS kit

| Name | Mutation | Exon |
| --- | --- | --- |
| Ex 18-mutant-1 | G719A | 18 |
| Ex 18-mutant-3 | G719C | 18 |
| Ex 19-mutant-1 | E746_A750del(1) | 19 |
| Ex 19-mutant-2 | E746_A750del(2) | 19 |
| Ex 19-mutant-3 | L747_P753>S | 19 |
| Ex 19-mutant-4 | E746_T751>I | 19 |
| Ex 19-mutant-5 | E746_T751del | 19 |
| Ex 19-mutant-6 | E746_T751>A | 19 |
| Ex 19-mutant-7 | E746_S752>A | 19 |
| Ex 19-mutant-8 | E746_S752>V | 19 |
| Ex 19-mutant-9 | E746_S752>D | 19 |
| Ex 19-mutant-10 | L747_A750>P | 19 |
| Ex 19-mutant-11 | L747_T751>Q | 19 |
| Ex 19-mutant-12 | L747_E749del | 19 |
| Ex 19-mutant-13 | L747_T751del | 19 |
| Ex 19-mutant-14 | L747_S752del | 19 |
| Ex 19-mutant-15 | L747_A750>P | 19 |
| Ex 19-mutant-16 | L747_P753>Q | 19 |
| Ex 19-mutant-17 | L747_T751>S | 19 |
| Ex 19-mutant-18 | L747_T751del | 19 |
| Ex 19-mutant-19 | L747_T751>P | 19 |
| Ex 19-mutant-20 | L747_T751del | 19 |
| Ex 19-mutant-21 | L747_S752>Q | 19 |
| Ex 19-mutant-22 | E746_T751>V | 19 |
| Ex 19-mutant-23 | E746_T751>T | 19 |
| Ex 19-mutant-24 | L747_A750>P | 19 |
| Ex 19-mutant-25 | L747_K754>QL | 19 |
| Ex 19-mutant-26 | E746_K754>EQHL | 19 |
| Ex 19-mutant-27 | E746_S752>EQ | 19 |
| Ex 19-mutant-28 | E746_A750>QP | 19 |
| Ex 19-mutant-29 | E746_T751>Q | 19 |
| Ex 20-mutant-1 | T790M | 20 |
| Ex 20-mutant-2 | S768I | 20 |
| Ex 20-mutant-3 | H773_V774insH | 20 |
| Ex 20-mutant-4 | D770_N771insG | 20 |
| Ex 20-mutant-5 | V769_D770insASV | 20 |
| Ex 20-mutant-8 | D770_N771insSVD | 20 |
| Ex 20-mutant-9 | D770ASVD | 20 |
| Ex 20-mutant-10 | H773_V774insNPH | 20 |
| Ex 21-mutant-1 | L858R | 21 |
| Ex 21-mutant-2 | L861Q | 21 |
