# Supplementary material for: Clinical validation of a highly sensitive assay to detect EGFR mutations in plasma cell-free DNA from patients with advanced lung adenocarcinoma
Source: PLoS One. 2017 Aug 22;12(8):e0183331. doi: 10.1371/journal.pone.0183331 (PMC5568724; doi:10.1371/journal.pone.0183331)
Supplement: S3 Table — EGFR, epidermal growth factor receptor; E19Del: exon 19 deletion. (DOCX) [file pone.0183331.s003.docx]

S3 Table EGFR mutation status in plasma and matched tumor tissue samples

|  | Tumor tissue | | | | | | | |  |
| --- | --- | --- | --- | --- | --- | --- | --- | --- | --- |
| Plasma | E19Dels | L858R | G719X | 20-ins | E19Dels +T790M | L858R+T790M | E19Dels +L858R | Wild | Total |
| E19Dels | 25 | 0 | 0 | 0 | 0 | 0 | 0 | 0 | 25 |
| L858R | 0 | 19 | 0 | 0 | 0 | 1 | 1 | 0 | 21 |
| 20-ins | 0 | 0 | 0 | 1 | 0 | 0 | 0 | 0 | 1 |
| E19Dels +T790M | 0 | 0 | 0 | 0 | 2 | 0 | 0 | 0 | 2 |
| L858R+T790M | 0 | 1 | 0 | 0 | 0 | 0 | 0 | 0 | 1 |
| Wild | 6 | 3 | 1 | 0 | 0 | 2 | 0 | 47 | 59 |
| Total | 31 | 23 | 1 | 1 | 2 | 3 | 1 | 47 | 109 |
