# Supplementary material for: Clinical validation of a highly sensitive assay to detect EGFR mutations in plasma cell-free DNA from patients with advanced lung adenocarcinoma
Source: PLoS One. 2017 Aug 22;12(8):e0183331. doi: 10.1371/journal.pone.0183331 (PMC5568724; doi:10.1371/journal.pone.0183331)
Supplement: S1 File — (DOCX) [file pone.0183331.s004.docx]

**Supplementary Methods**

**Assessing the analytical sensitivity and specificity of the ADx-SuperARMS EGFR** **assay**

Three major EGFR mutation subtypes namely exon 19 deletion (E19Del), L858R, and T790M were included for the assessment of the analytical sensitivity and specificity of the new method. DNA samples were prepared by using serial dilution method where mutant genomic DNA derived from tumor cell lines (H1650 for E19Del, H1975 for L858R and T790M) and normal genomic DNA were mixed. The total inputting DNA was fixed at 3000 copies of genome equivalents (GEs) for each reaction, and a series of percentages of mutant DNA in total DNA, including 5%, 1%, 0.2% and 0.1% were tested to define the analytical sensitivity. Wild type genomic DNA samples were tested for confirming the analytical specificity. There were three repeats for each data point.
