# Supplementary material for: Clinical validation of a highly sensitive assay to detect EGFR mutations in plasma cell-free DNA from patients with advanced lung adenocarcinoma
Source: PLoS One. 2017 Aug 22;12(8):e0183331. doi: 10.1371/journal.pone.0183331 (PMC5568724; doi:10.1371/journal.pone.0183331)
Supplement: S1 STROBE Checklist — (DOCX) [file pone.0183331.s005.docx]

STROBE Statement—checklist of items that should be included in reports of observational studies

|  | Item No. | Recommendation | Page  No. | Relevant text from manuscript |
| --- | --- | --- | --- | --- |
| **Title and abstract** | 1 | (*a*) Indicate the study’s design with a commonly used term in the title or the abstract | 2-3 |  |
|  |  | (*b*) Provide in the abstract an informative and balanced summary of what was done and what was found | 2-3 | ADx-SuperARMS EGFR assay is likely to be a highly sensitive and specific method to noninvasively detect plasma EGFR mutations of patients with advanced lung adenocarcinoma. |
| Introduction | | | |  |
| Background/rationale | 2 | Explain the scientific background and rationale for the investigation being reported | 4 | In recent years, EGFR-TKIs including Gefitinib, Erlotinib,  Icotinib, and Afatinib have been successfully developed and demonstrated  much higher response rate and less side effect than chemotherapy in the   treatment of patients with advanced NSCLC with sensitizing EGFR  mutations. EGFR mutation detection to select right patients for  EGFR-TKI treatment is critical for clinical practice |
| Objectives | 3 | State specific objectives, including any prespecified hypotheses | 5 | In the present study, we implemented a clinical study to evaluate the performance of this new method in testing clinical plasma samples as well as in predicting the efficacy of EGFR-TKIs treatment. |
| Methods | | | |  |
| Study design | 4 | Present key elements of study design early in the paper | 5 | To evaluate the performance of the new method in testing clinical plasma samples. In addition, the clinical value of the  plasma EGFR mutation detection by the new method in predicting the  efficacy of EGFR-TKIs treatment was also explored. |
| Setting | 5 | Describe the setting, locations, and relevant dates, including periods of recruitment, exposure, follow-up, and data collection | 5 | Three major EGFR mutation types including exon 19 deletion  (E19Del), L858R, and T790M were included for verification of analytical  sensitivity and specificity of the new method. |
| Participants | 6 | (*a*) *Cohort study*—Give the eligibility criteria, and the sources and methods of selection of participants. Describe methods of follow-up  *Case-control study*—Give the eligibility criteria, and the sources and methods of case ascertainment and control selection. Give the rationale for the choice of cases and controls  *Cross-sectional study*—Give the eligibility criteria, and the sources and methods of selection of participants | 5 | This study was conducted in the Department of  Pulmonary and Critical Care Medicine, the first affiliated hospital of  Wenzhou Medical University in Zhejiang, China. Patients were enrolled from 1 August 2016 to 1 January 2017. |
|  |  | (*b*) *Cohort study*—For matched studies, give matching criteria and number of exposed and unexposed  *Case-control study*—For matched studies, give matching criteria and the number of controls per case | 5 | Inclusion criteria: 1) pathologically  confirmed lung adenocarcinoma; 2) advanced clinical stage (stage IIIB or  IV); 3) newly diagnosed or progressive disease (PD) after EGFR-TKIs or  recurrence after surgery; 4) a subsequent treatment did not begin at the time  of sample collection; 5) valid tissue and blood specimens available and the  interval between tissue collection and blood collection was less than 14  days. |
| Variables | 7 | Clearly define all outcomes, exposures, predictors, potential confounders, and effect modifiers. Give diagnostic criteria, if applicable | 7-8 | the Response Evaluation  Criteria In Solid Tumors version 1.1 |
| Data sources/ measurement | 8* | For each variable of interest, give sources of data and details of methods of assessment (measurement). Describe comparability of assessment methods if there is more than one group | 7-8 | the Response Evaluation  Criteria In Solid Tumors version 1.1 |
| Bias | 9 | Describe any efforts to address potential sources of bias | 8 |  |
| Study size | 10 | Explain how the study size was arrived at | 5 | This study was conducted in the Department of  Pulmonary and Critical Care Medicine, the first affiliated hospital of Wenzhou Medical University in Zhejiang, China. Patients were enrolled  from 1 August 2016 to 1 January 2017. |

Continued on next page

| Quantitative variables | 11 | Explain how quantitative variables were handled in the analyses. If applicable, describe which groupings were chosen and why | 7-8 | the Response Evaluation  Criteria In Solid Tumors version 1.1 |
| --- | --- | --- | --- | --- |
| Statistical methods | 12 | (*a*) Describe all statistical methods, including those used to control for confounding | 7-8 | EGFR mutation status in tumor tissues were used as standard  reference for the calculation of the sensitivity and specificity plasma EGFR   mutation detection. To assess the value of EGFR mutations in ctDNA in  predicting efficacy of first generation of EGFR-TKIs, the objective  response rate (ORR) were measured according to the Response Evaluation  Criteria In Solid Tumors version 1.1. |
|  |  | (*b*) Describe any methods used to examine subgroups and interactions | 8 |  |
|  |  | (*c*) Explain how missing data were addressed | 8 |  |
|  |  | (*d*) *Cohort study*—If applicable, explain how loss to follow-up was addressed  *Case-control study*—If applicable, explain how matching of cases and controls was addressed  *Cross-sectional study*—If applicable, describe analytical methods taking account of sampling strategy | 8 |  |
|  |  | (*e*) Describe any sensitivity analyses | 7-8 |  |
| Results | | | | |
| Participants | 13* | (a) Report numbers of individuals at each stage of study—eg numbers potentially eligible, examined for eligibility, confirmed eligible, included in the study, completing follow-up, and analysed | 8 | A total of 109 patients met the criteria and were prospectively enrolled   into the study. |
|  |  | (b) Give reasons for non-participation at each stage | 8 | A total of 109 patients met the criteria and were prospectively enrolled   into the study. |
|  |  | (c) Consider use of a flow diagram |  |  |
| Descriptive data | 14* | (a) Give characteristics of study participants (eg demographic, clinical, social) and information on exposures and potential confounders | 8-9 | Table 1 Clinical characteristics of 109 patients with lung adenocarcinoma |
|  |  | (b) Indicate number of participants with missing data for each variable of interest | 9 |  |
|  |  | (c) *Cohort study*—Summarise follow-up time (eg, average and total amount) | 12-13 |  |
| Outcome data | 15* | *Cohort study*—Report numbers of outcome events or summary measures over time | 12-13 | 27 (64.3%) patients achieved a partial response (PR), 14 (33.3%) sustained disease, and 1 (2.4%) progression disease. The objective response rate (ORR) and disease control rate (DCR) were 64.3% (95% CI, 48.0–78.0%) and 97.6% (95% CI, 87.0–100.0%), respectively. An objective response was seen in 23 of 35 patients who were EGFR mutation-positive in both tumor and matched plasma, with an ORR of 65.7% (95% CI, 48.0–81.0%) and DCR of 97.1% (95% CI, 85.0–100.0%) (23 with PR, 11 with SD, 1 with PD). An ORR of 57.1% (4/7, 95% CI, 18.0–90.0%) was observed among the 7 patients who were mutation-positive in tumor but mutation-negative in plasma. |
|  |  | *Case-control study—*Report numbers in each exposure category, or summary measures of exposure |  |  |
|  |  | *Cross-sectional study—*Report numbers of outcome events or summary measures |  |  |
| Main results | 16 | (*a*) Give unadjusted estimates and, if applicable, confounder-adjusted estimates and their precision (eg, 95% confidence interval). Make clear which confounders were adjusted for and why they were included | 13 | 27 (64.3%) patients achieved a partial response (PR), 14 (33.3%) sustained disease, and 1 (2.4%) progression disease. The objective response rate (ORR) and disease control rate (DCR) were 64.3% (95% CI, 48.0–78.0%) and 97.6% (95% CI, 87.0–100.0%), respectively. An objective response was seen in 23 of 35 patients who were EGFR mutation-positive in both tumor and matched plasma, with an ORR of 65.7% (95% CI, 48.0–81.0%) and DCR of 97.1% (95% CI, 85.0–100.0%) (23 with PR, 11 with SD, 1 with PD). An ORR of 57.1% (4/7, 95% CI, 18.0–90.0%) was observed among the 7 patients who were mutation-positive in tumor but mutation-negative in plasma. |
|  |  | (*b*) Report category boundaries when continuous variables were categorized |  |  |
|  |  | (*c*) If relevant, consider translating estimates of relative risk into absolute risk for a meaningful time period |  |  |

Continued on next page

| Other analyses | 17 | Report other analyses done—eg analyses of subgroups and interactions, and sensitivity analyses | 10-11 | Firstly, the analytical sensitivity and specificity of the ADx-SuperARMS assays for EGFR E19Dels, L858R, and T790M mutations were evaluated by using a series of mock DNA samples (S1 File). |
| --- | --- | --- | --- | --- |
| Discussion | | | | |
| Key results | 18 | Summarise key results with reference to study objectives | 15 | In conclusion, ADx-SuperARMS is likely to provide a highly sensitive and specific noninvasive detection of EGFR mutations in clinical blood samples from advanced lung adenocarcinoma patients to guide target therapy as well as to monitor drug resistance, and the EGFR mutation status detected in plasma with ADx-SuperARMS could predict the efficacy of EGFR-TKIs treatment. Hence, EGFR blood testing with ADx-SuperARMS offers a good option to address the unmet clinical needs. |
| Limitations | 19 | Discuss limitations of the study, taking into account sources of potential bias or imprecision. Discuss both direction and magnitude of any potential bias | 15 | Additional clinical study is ongoing on more patients who have progressed disease after EGFR-TKIs treatment.  As the study cohort is relatively small in this study, further investigation of larger patient population is needed to confirm the findings from this study. |
| Interpretation | 20 | Give a cautious overall interpretation of results considering objectives, limitations, multiplicity of analyses, results from similar studies, and other relevant evidence | 15 | Previous studies reported significant correlations between ctDNA EGFR mutation status and the clinical response to EGFR-TKIs[23, 24, 29]. Here in our study, the ORR of EGFR-TKIs treatment in plasma EGFR mutation positive patients was very close to the ORR in tissue EGFR mutation positive patients (65.7% vs. 64.3%), and it was better than in plasma EGFR negative cohort (65.7% vs 57.1%, Figure 2), suggesting that the blood testing with ADx-SuperARMS could predict the patients’ benefit from EGFR-TKIs treatment. The progression-free survival and overall survival of patients during EGFR-TKIs treatment have not been achieved and the clinical follow-up observation is still ongoing. |
| Generalisability | 21 | Discuss the generalisability (external validity) of the study results | 14-15 | Molecular testing of ctDNA is reported to provide a dynamic monitoring of acquired resistance to first generation EGFR-TKIs.  In conclusion, ADx-SuperARMS is likely to provide a highly sensitive and specific noninvasive detection of EGFR mutations in clinical blood samples from advanced lung adenocarcinoma patients to guide target therapy as well as to monitor drug resistance, and the EGFR mutation status detected in plasma with ADx-SuperARMS could predict the efficacy of EGFR-TKIs treatment. Hence, EGFR blood testing with ADx-SuperARMS offers a good option to address the unmet clinical needs. |
| Other information | |  | | |
| Funding | 22 | Give the source of funding and the role of the funders for the present study and, if applicable, for the original study on which the present article is based |  | This study was funded by grants from the Natural Science Foundation of Zhejiang Province[LY15H010007 to Yuping Li] and the Wenzhou Municipal Science and Technology Bureau[ZH2017001 to Yuping Li]. |

*Give information separately for cases and controls in case-control studies and, if applicable, for exposed and unexposed groups in cohort and cross-sectional studies.

**Note:** An Explanation and Elaboration article discusses each checklist item and gives methodological background and published examples of transparent reporting. The STROBE checklist is best used in conjunction with this article (freely available on the Web sites of PLoS Medicine at http://www.plosmedicine.org/, Annals of Internal Medicine at http://www.annals.org/, and Epidemiology at http://www.epidem.com/). Information on the STROBE Initiative is available at www.strobe-statement.org.
